# Supplementary material for: Macroscopic Polarization Change via Electron Transfer in a Valence Tautomeric Cobalt Complex
Source: Nat Commun. 2020 Apr 24;11:1992. doi: 10.1038/s41467-020-15988-1 (PMC7181709; doi:10.1038/s41467-020-15988-1)
Supplement: Supplementary file 1 — Supplementary Information [file 41467_2020_15988_MOESM1_ESM.pdf]

Supplementary Information for

Macroscopic Polarization Change via Electron Transfer in a Valence Tautomeric Cobalt Complex

Wu et al.

Correspondence to: kanegawa@cm.kyushu-u.ac.jp; sato@cm.kyushu-u.ac.jp.

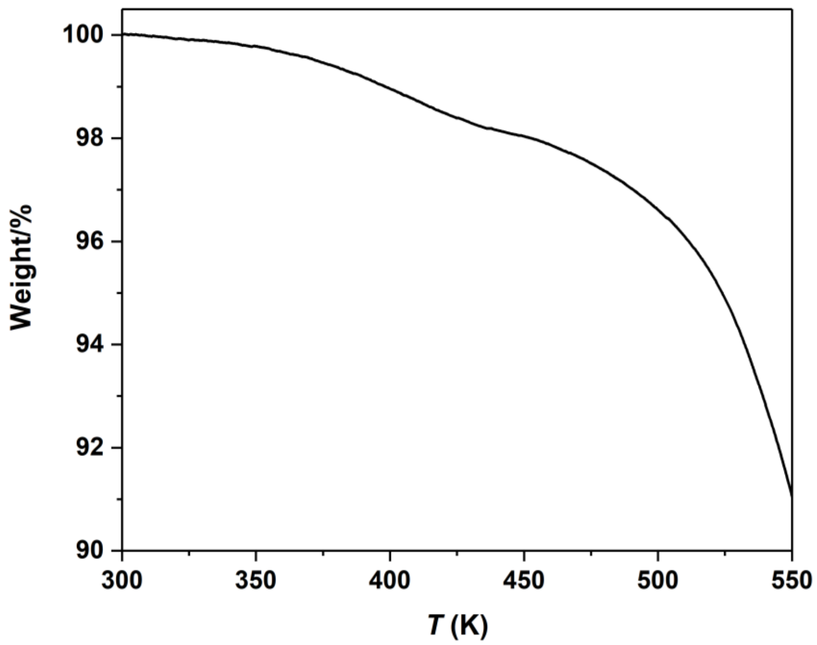

Supplementary Figure 1. Thermogravimetric analysis of **1**·0.5EtOH. The desolvation process at high temperature (> 420 K) is accompanied by the substantial weight loss possibly due to decomposition. It should be noted that the SQUID measurement was performed in a low-pressure environment, which allows desolvation at much lower temperature.

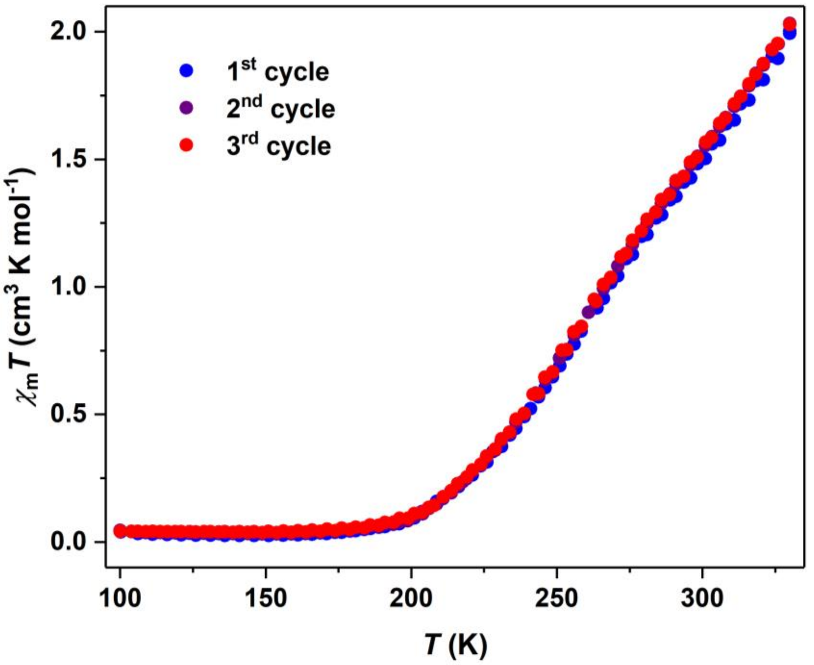

Supplementary Figure 2. Magnetic measurement performed between 100 and 330 K with a He gas flow. Notably, the measurement conditions are the same with those used for the pyroelectric measurement in the main text. No significant solvent effect was found (ΔT < 4 K), confirming the rationality behind the pyroelectric measurement conditions.

Supplementary Table 1. Crystallographic data and structure refinement parameters of **1**·0.5EtOH.

Temperature	123 K	183 K	293 K	393 K	123 K-2
Formula	$C_{62}H_{94}Cl_2Co_2N_8O_{13}$	$C_{62}H_{94}Cl_2Co_2N_8O_{13}$	$C_{62}H_{94}Cl_2Co_2N_8O_{13}$	$C_{62}H_{94}Cl_2Co_2N_8O_{13}$	$C_{62}H_{94}Cl_2Co_2N_8O_{13}$
Formula weight	1348.21	1348.21	1348.21	1348.21	1348.21
Crystal system	monoclinic	monoclinic	monoclinic	monoclinic	monoclinic
Space group	$P2_1$	$P2_1$	$P2_1$	$P2_1$	$P2_1$
a / Å	10.6518(2)	10.6640(2)	10.8356(3)	10.9455(3)	10.6378(2)
b / Å	27.4074(5)	27.4695(4)	27.8843(6)	28.1029(6)	27.3549(4)
c / Å	11.6557(3)	11.6951(3)	11.7492(4)	11.9586(4)	11.6588(3)
α / °	90	90	90	90	90
β / °	111.165(3)	111.108(2)	111.196(4)	111.195(4)	111.233(2)
γ / °	90	90	90	90	90
V / Å^3	3173.2(1)	3196.0(1)	3309.8(2)	3429.6(2)	3162.4(1)
Z	2	2	2	2	2
D_c / g·cm^{-3}	1.411	1.402	1.353	0.993	1.415
F(000)	1428	1428	1428	1428	1428
GOF on F^2	1.032	1.000	1.037	0.993	1.040
R1,wR2 [$I > 2\sigma(I)$]	0.0325, 0.0785	0.0343, 0.0924	0.0408, 0.1104	0.0423, 0.1160	0.0366, 0.0924
R1,wR2 (all data)	0.0347, 0.0799	0.0384, 0.0949	0.0514, 0.1165	0.0559, 0.1242	0.0386, 0.0934
Friedel coverage	0.902	0.871	0.846	0.942	0.957
Flack parameter	-0.004(3)	-0.009(3)	-0.007(3)	-0.001(4)	0.000(3)
CCDC	1952513	1952512	1952514	1952515	1952516

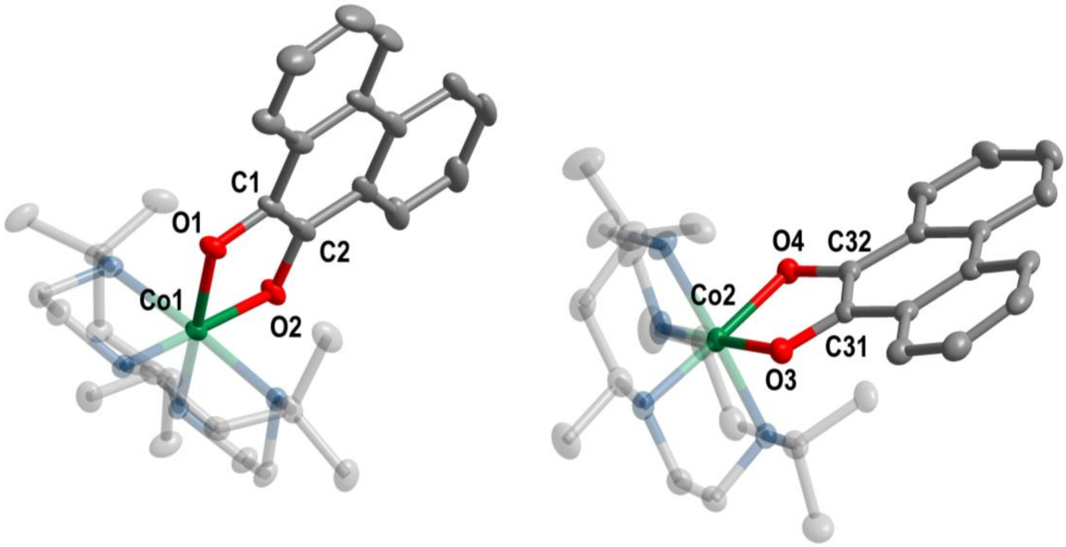

Supplementary Figure 3. Crystal structure of the pair of *Δ*-[Co(phendiox)(*SS*-cth)]$^{+}$ (Co1-centered motif) and *Λ*-[Co(phendiox)(*RR*-cth)]$^{+}$ (Co2-centered motif) in **1**·0.5EtOH at 123 K with atomic labels. Hydrogen atoms, anions and solvent molecules are omitted for clarity.

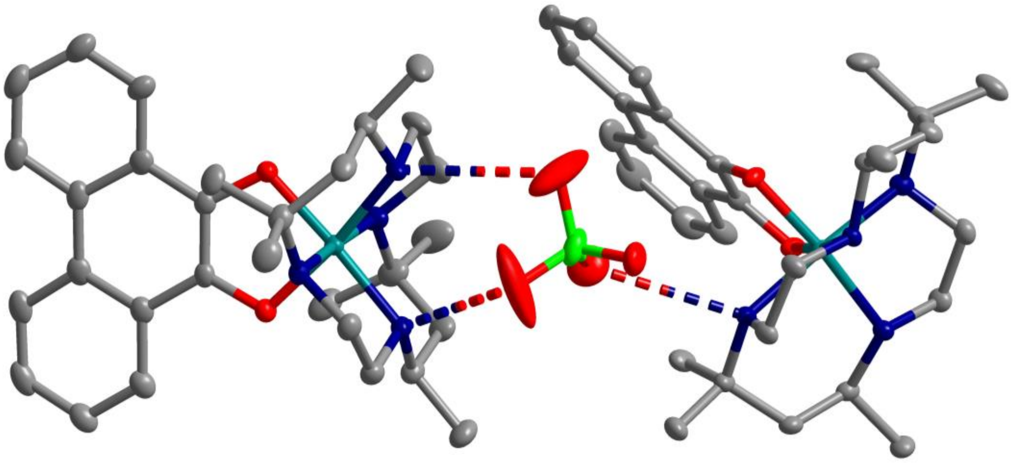

Supplementary Figure 4. Weak hydrogen bonds between perchlorate anion and the two racemic cth ligands at 123 K.

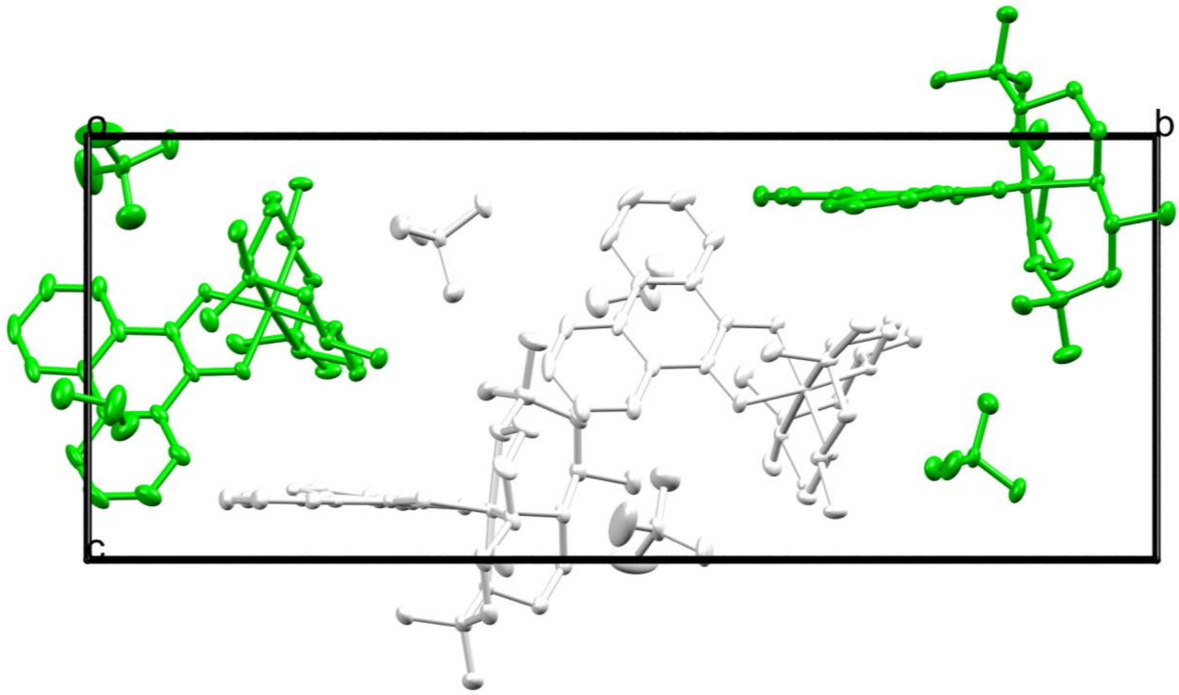

Supplementary Figure 5. Crystal packing viewed along the *a*-axis at 123 K. Asymmetric units are colored with respect to the two-fold screw operation.

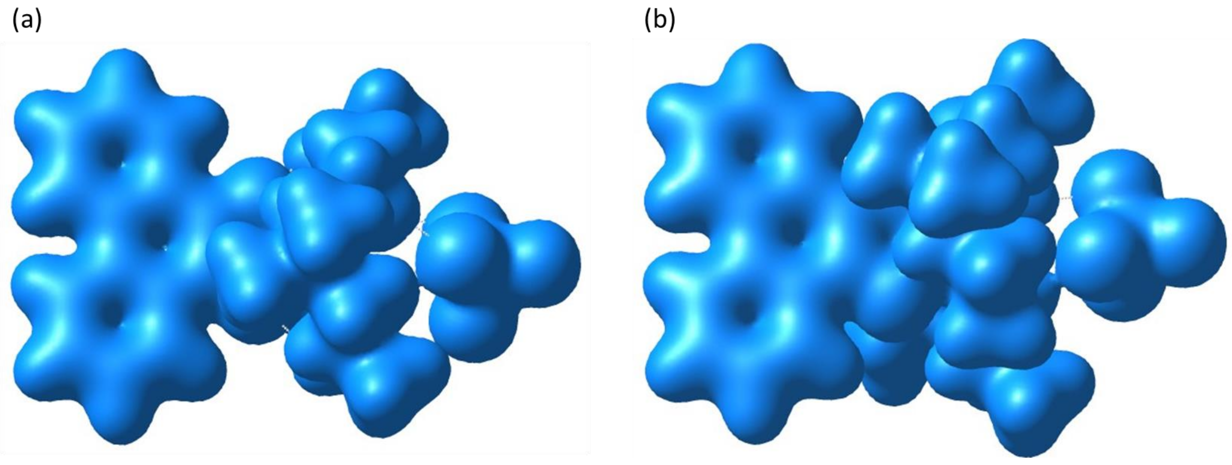

Δ-[Co(phendiox)(*SS*-cth)] (H-optimized) *Λ*-[Co(phendiox)(*RR*-cth)] (H-optimized)

Supplementary Figure 6. Contour surface of electronic densities of the two enantiomeric Co-centered motifs ((a) and (b) panels show the electronic densities of *Δ*- and *Λ*-enantiomers, respectively) with a contour value of 0.0205 e·Å^{-3}. Hydrogen atoms are optimized at the B3LYP-D3/6-311+G** level of theory. The electronic density from the perchlorate anion starts to overlap with the density from the N−H bond in *Λ*-enantiomer first, showing stronger steric and electronic effects that lead to the higher transition temperature of the *Λ*-enantiomer.

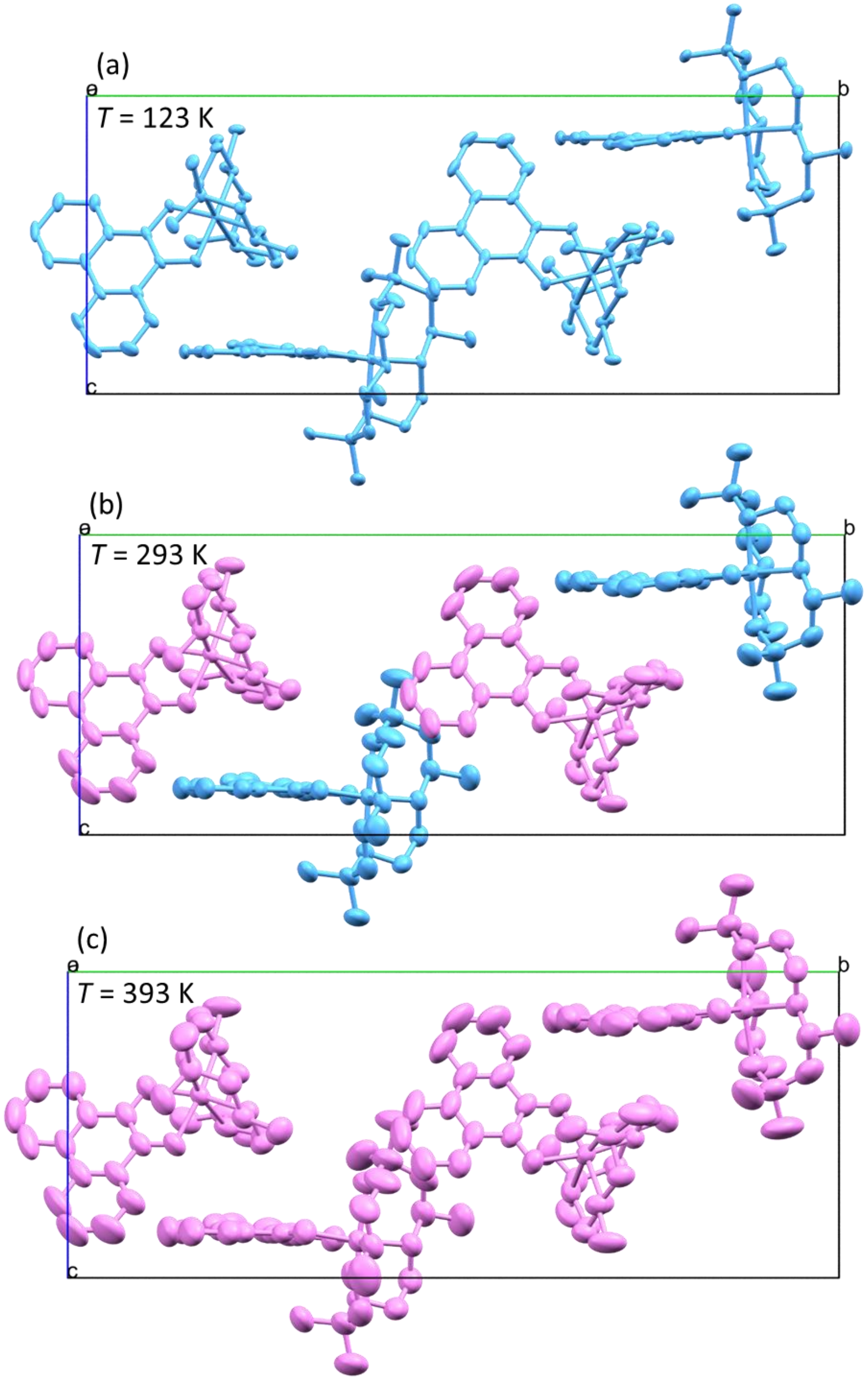

Supplementary Figure 7. Two-step valence tautomeric transition in a unit cell. The low-spin and high-spin Co-centered motifs are colored in blue and pink, respectively. One of the two crystallographically independent Co enantiomers exhibits valence tautomerism upon heating from 123 (a) to 293 K (b), followed by the valence tautomerism of the other enantiomer upon further heating (c). Hydrogen atoms, anions, and solvent molecules are omitted for clarity.

Supplementary Table 2. Selected coordination and ligand bond lengths (Å) for **1**·0.5EtOH (atomic number labels are listed in Figure S4).

Temperature	123 K	183 K	293 K	393 K	123 K-2
Δ-Co1-O	1.890(2)	1.890(2)	2.065(3)	2.098(3)	1.888(3)
	1.887(2)	1.886(2)	2.058(3)	2.083(4)	1.883(3)
Δ-Co1-N	2.009(3)	2.008(2)	2.128(3)	2.142(4)	2.006(3)
	2.031(3)	2.030(3)	2.175(3)	2.198(4)	2.030(3)
	2.002(3)	2.009(2)	2.127(3)	2.156(4)	2.008(3)
	2.016(3)	2.018(3)	2.153(3)	2.179(4)	2.016(3)
O1-C1	1.355(4)	1.349(4)	1.297(5)	1.277(6)	1.353(5)
O2-C2	1.358(4)	1.355(4)	1.289(5)	1.298(6)	1.353(5)
C1-C2	1.359(6)	1.365(5)	1.410(6)	1.407(8)	1.358(6)
Δ-Co2-O	1.892(2)	1.891(2)	1.901(3)	2.064(4)	1.891(3)
	1.897(3)	1.894(2)	1.900(3)	2.072(4)	1.896(3)
Δ-Co2-N	2.014(3)	2.015(3)	2.031(3)	2.157(5)	2.016(3)
	1.997(3)	1.998(3)	2.014(3)	2.108(5)	2.001(4)
	2.025(3)	2.026(3)	2.033(4)	2.180(5)	2.023(4)
	2.003(3)	2.002(3)	2.019(3)	2.127(5)	2.003(3)
O3-C31	1.352(4)	1.348(4)	1.348(5)	1.285(7)	1.348(5)
O4-C32	1.348(4)	1.344(4)	1.354(5)	1.281(7)	1.349(5)
C31-C32	1.375(5)	1.371(4)	1.370(6)	1.424(8)	1.372(6)

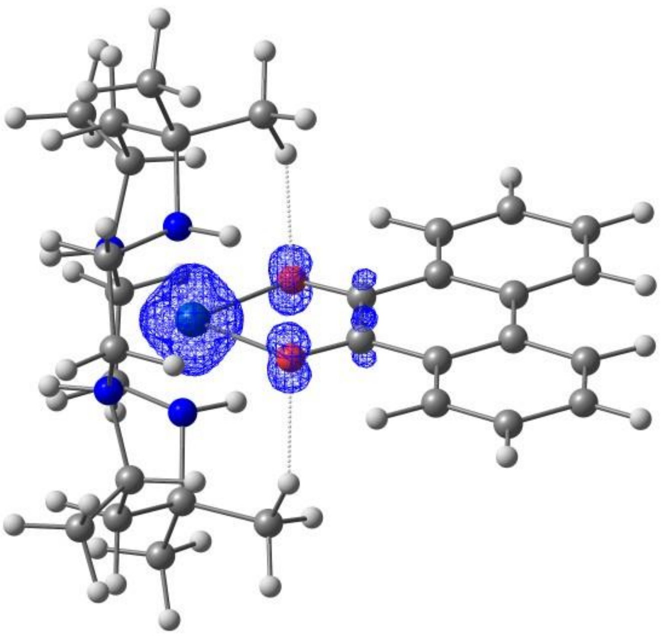

Supplementary Figure 8. Spin density of $[Co^{II}(phenSq)(cth)]^+$ in the quintet state. The contour value of the density envelope is 0.025 Å^{-3}.

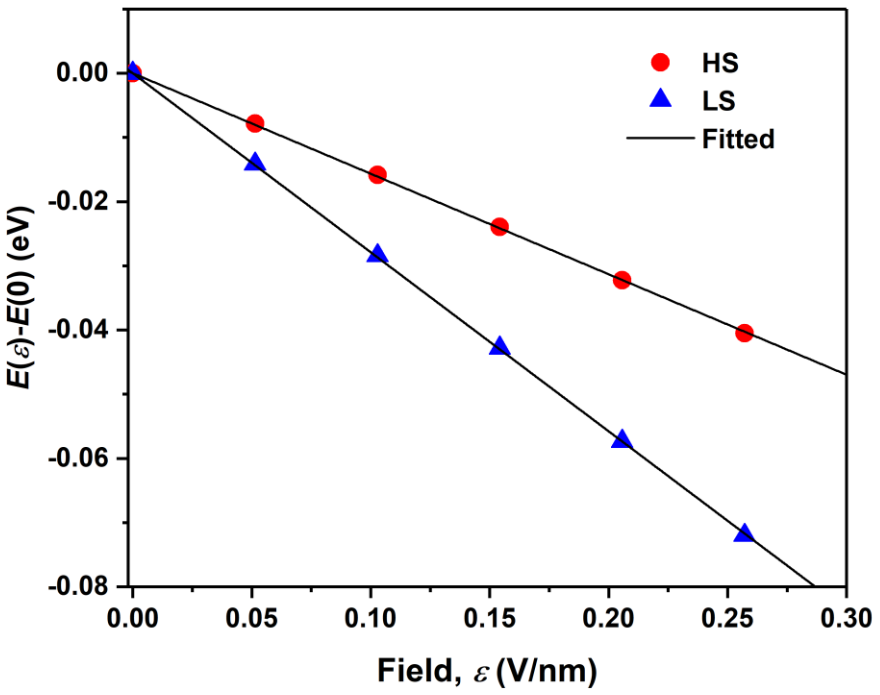

Supplementary Figure 9. Permanent dipole moments calculated from the first-order Stark effect. The linear coefficients are −0.1566(8) eV·nm·V^{-1} (corresponding to 7.52 Debye) for the high-spin motif and −0.2788(6) eV·nm·V^{-1} (corresponding to 13.39 Debye) for the low-spin motif, respectively.

Supplementary Table 3. Calculation of polarization change during the electron transfer process.

T	θ_1/º	θ_2/º	μ_{total}/D	V_{cell}/Å^3	Δ*P*/ μC·cm^{-2}
**123 K**	27.42	25.52	23.54	3173.2	0
**183 K**	27.06	25.29	23.60	3196.0	-0.010
**293 K**	28.62	25.41	18.26	3309.8	-0.634
**393 K**	28.10	25.09	13.00	3429.6	-1.211

1) θ_1, θ_2 is the angle between the dipole moments of the Co1- and Co2-centered motifs and the crystalline *b*-axis calculated from single-crystal measurements;

2) μ_{total} is the sum of dipole moments of the Co1- and Co2-centered motifs (obtained from DFT calculations) projected on the crystalline *b*-axis; $\mu_{total} = \mu_{Co1}\cos\theta_1 + \mu_{Co2}\cos\theta_2$;

3) V_{cell} is the volume of the unit cell at the given temperature;

4) Δ*P* is the polarization change; $\Delta P(T) = \mu_{total}(T)/V_{cell}(T) - \mu_{total}(123\ \text{K})/V_{cell}(123\ \text{K})$.

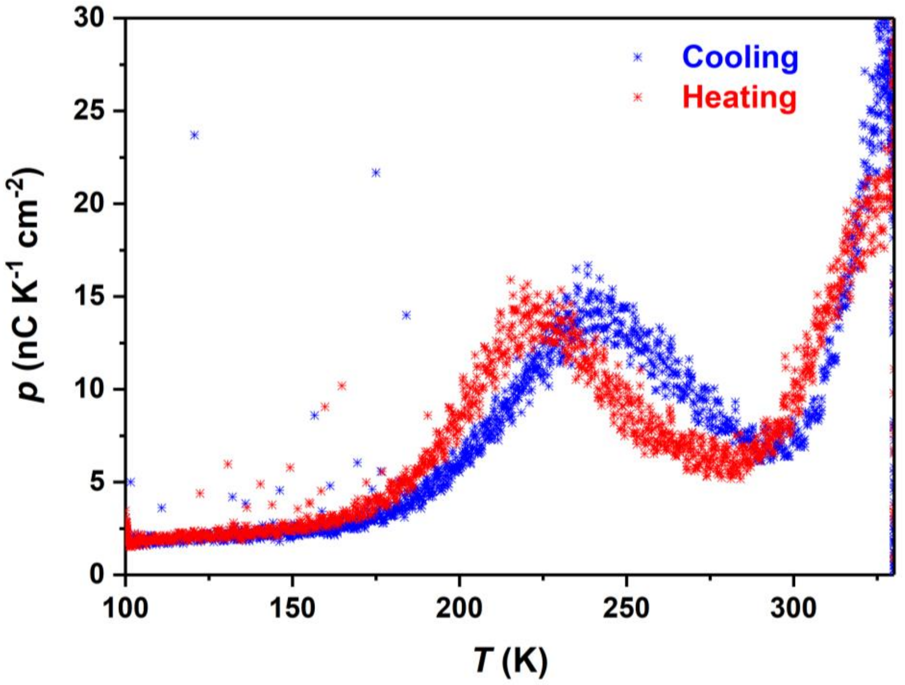

Supplementary Figure 10. Pyroelectric coefficient (*p*) on a single crystal of **1**·0.5EtOH between 100 and 330 K with a sweep rate of 10 K·min^{-1}. The results are almost identical to those obtained with a sweep rate of 5 K·min^{-1} in the main text.

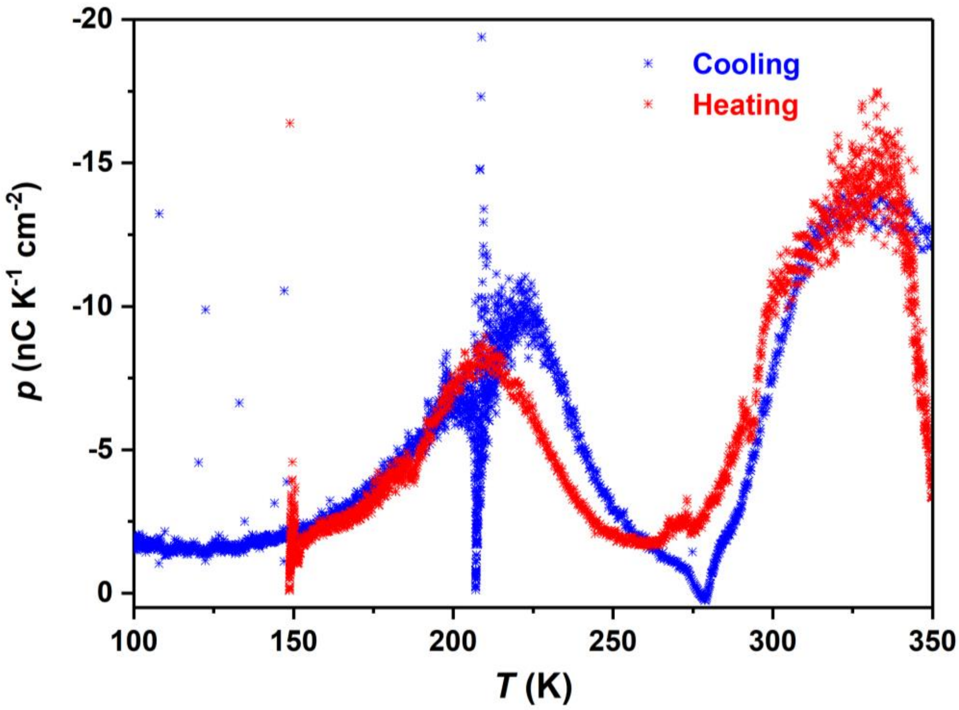

Supplementary Figure 11. Pyroelectric coefficient (*p*) on a single crystal of **1**·0.5EtOH between 100 and 350 K measured in the homemade shield box.

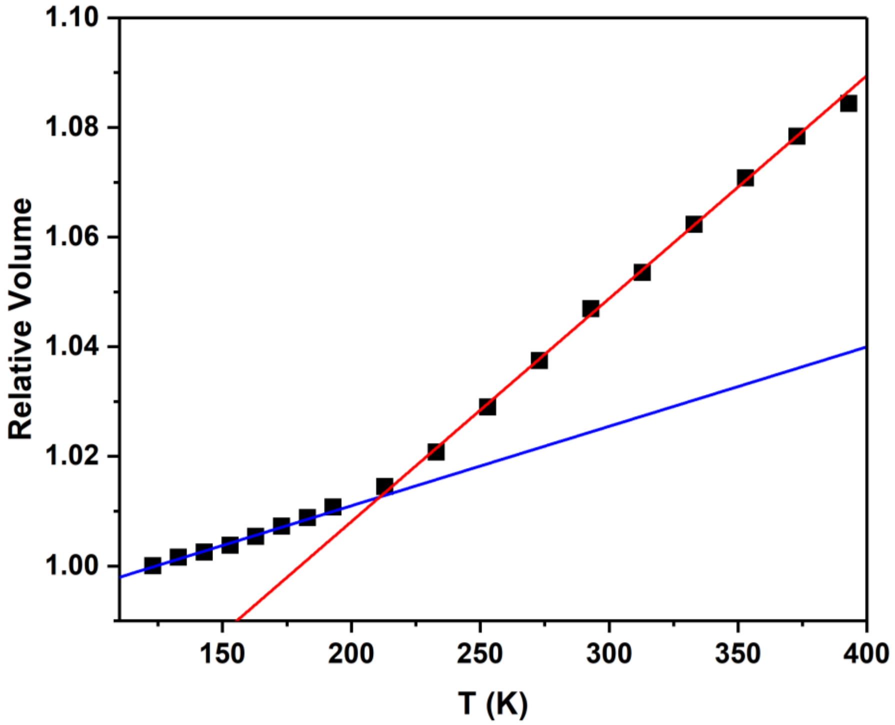

Supplementary Figure 12. Thermal expansion of the lattice volume between 123 and 393 K. The volumetric thermal expansion coefficient during the electron transfer process (407(5)×10^{-6} K^{-1}) is much larger than that below the transition point (145(6)×10^{-6} K^{-1}).

Supplementary Table 4. Isotropic displacement of ethanol molecule in **1**·0.5EtOH.

Temperature	123 K	183 K	293 K	393 K	123 K-2
C(-CH_3)	0.060(2)	0.091(2)	0.218(6)	0.36(2)	0.078(2)
C(-CH_2-)	0.069(2)	0.102(3)	0.193(5)	0.29(1)	0.091(3)
O(-OH)	0.052(1)	0.099(2)	0.334(8)	0.89(5)	0.072(2)

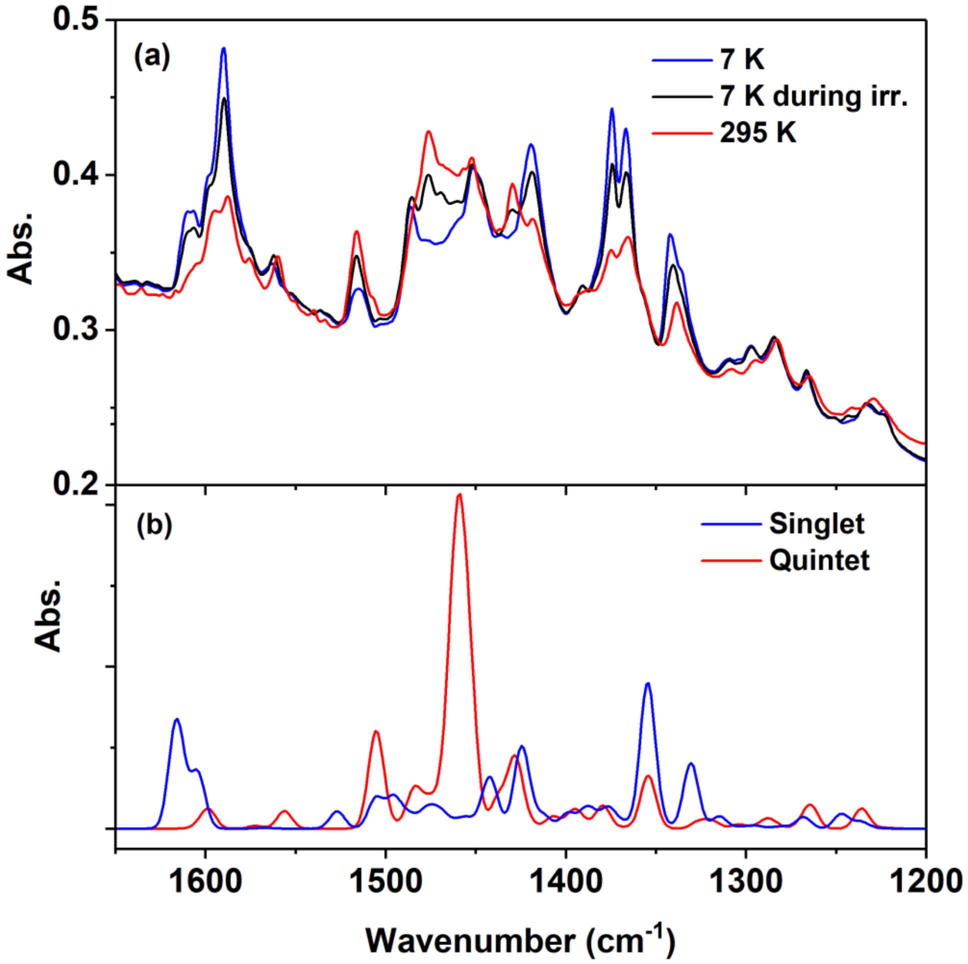

Supplementary Figure 13. (a) IR absorption spectra between 1200 and 1650 cm^{-1} at 7 K and room temperature, and during light irradiation at 7 K; (b) the calculated absorption spectra for the singlet and quintet states (bottom, the frequency scaling factor is 0.98). To remove the temperature-dependent background effect, the absorption at 1700 cm^{-1} was set to be the same for the different measurement conditions. Temperature-induced changes were observed in the IR absorption spectra of **1**·0.5EtOH at 7 and 295 K, which correspond to the states before and intermediate of the electron transfer process, respectively. According to theoretical calculation, the IR absorption bands at 1365 and 1374 cm^{-1}, which decrease upon heating, are assigned to the in-phase C–O stretching coupled with C=C stretching and C–H bending of phenCat^{2-}, whereas the corresponding vibrational band of $(phenSq)^{-}$ is observed at 1476 cm^{-1}, which increases upon heating.

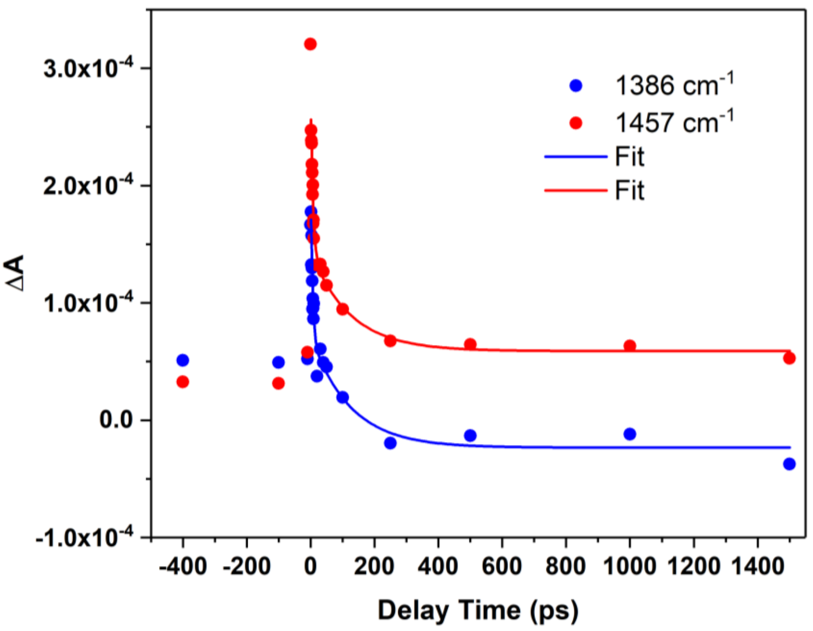

Supplementary Figure 14. Temporal profiles of transient IR absorption intensities at 1386 and 1457 cm^{-1}. Solid lines represent the fitting results with the double exponential law. The fitting relaxation times are τ_1 = 6 ps, τ_2 = 124 ps for 1386 cm^{-1} and τ_1 = 7 ps, τ_2 = 124 ps for 1457 cm^{-1}.

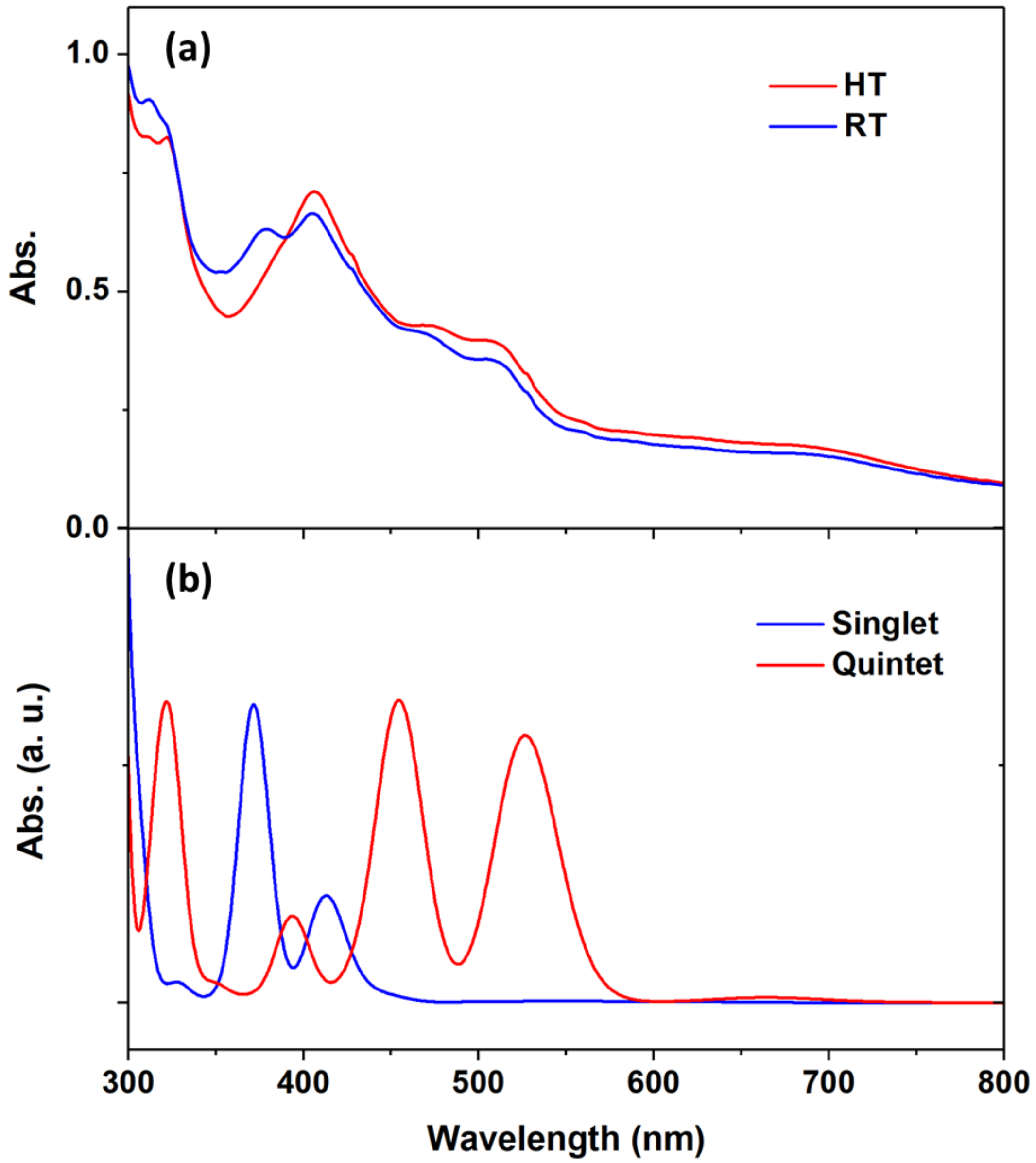

Supplementary Figure 15. (a) UV-vis absorption spectra between 300 and 800 nm at room temperature and ca. 400 K; (b) the calculated absorption spectra for the singlet and quintet states. The energy scaling factor is 0.90. The main absorption band centered at 378 nm at low temperature and the band centered at 406 nm at high temperature are assigned to the π−π* transition of the catechol-form ligand, based on the charge density difference between the ground and the excited states. A similar absorption peak was found in the reference compound, [Ga(phenCat)(*rac*-cth)](PF_6), in the same region, confirming the pure ligand origin of band centered at 378 nm for **1**·0.5EtOH.

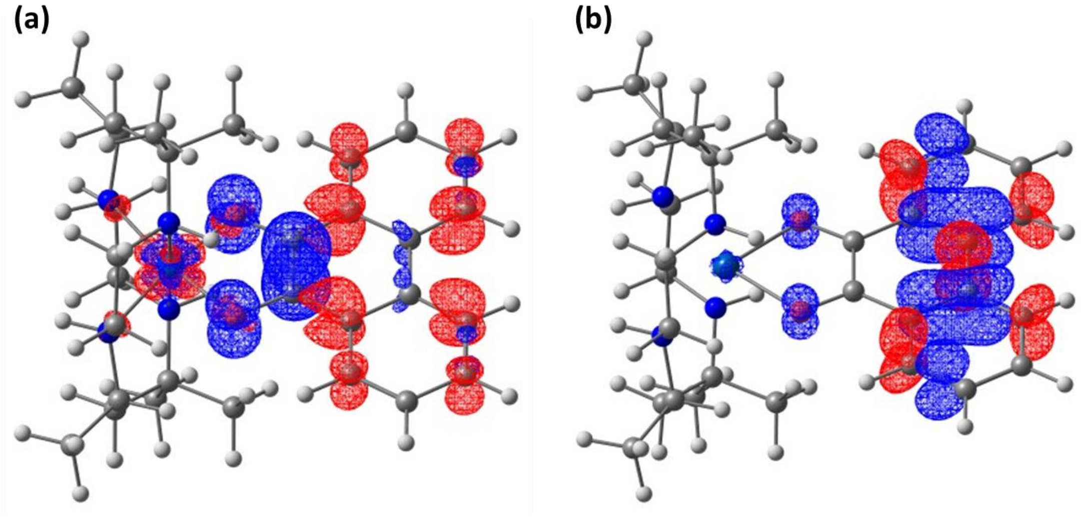

Supplementary Figure 16. Charge density differences between the excited and ground states in the closed-shell singlet state ($[Co^{III}(phenCat)(cth)]^{+}$) for transition at 380 nm (a) and the quintet state ($[Co^{II}(phenSq)(cth)]^{+}$) for transition at 396 nm (b). Red (increase) and blue (decrease) envelopes represent the change in the electronic density, which clearly exhibits a π-π* transition feature. The contour value of the density envelope is 0.0015 $Å^{-3}$. Notably, the charge density difference was also found on Co(III) in the closed-shell singlet state (left panel). However, it originates from a stronger overlap between the 3d orbitals of Co(III) ion and the ligand molecular orbitals.

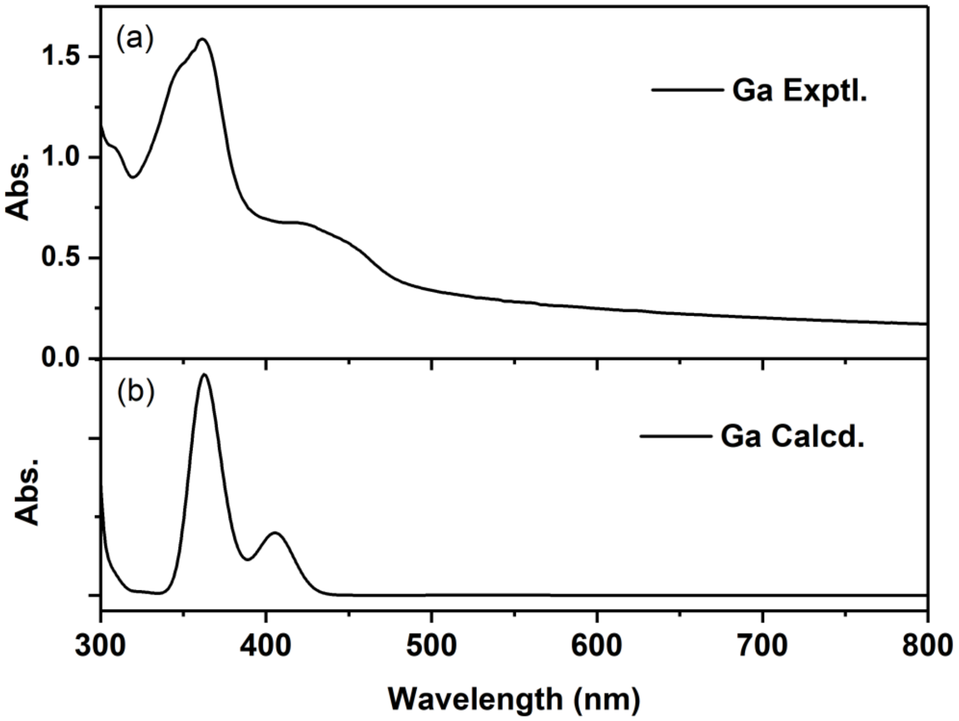

Supplementary Figure 17. Solid-state UV-vis absorption spectra of the Ga^{III} analogue ($[Ga(phenCat)(\textit{rac}\text{-}cth)](PF_6)$) compared with the calculated spectrum ((a) and (b) panels show the experimental and calculated spectra, respectively). The energy scaling factor is 0.90.

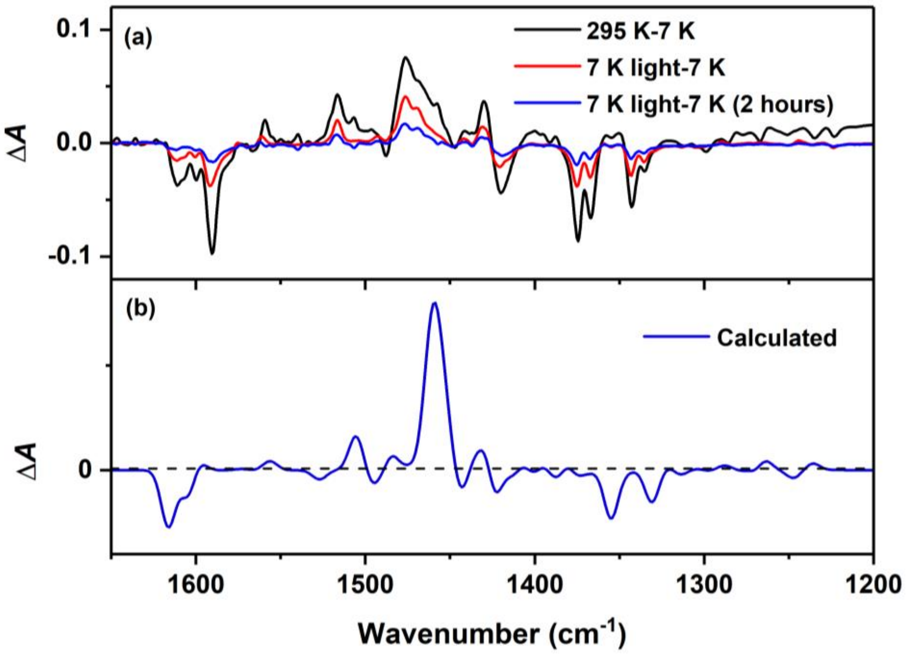

Supplementary Figure 18. (a) Difference between absorption spectra obtained at room temperature, at 7 K during light excitation, and at 7 K after two-hour relaxation with the spectrum at 7 K; (b) calculated difference of the IR intensities between the quintet and singlet states (the frequency scaling factor is 0.98).

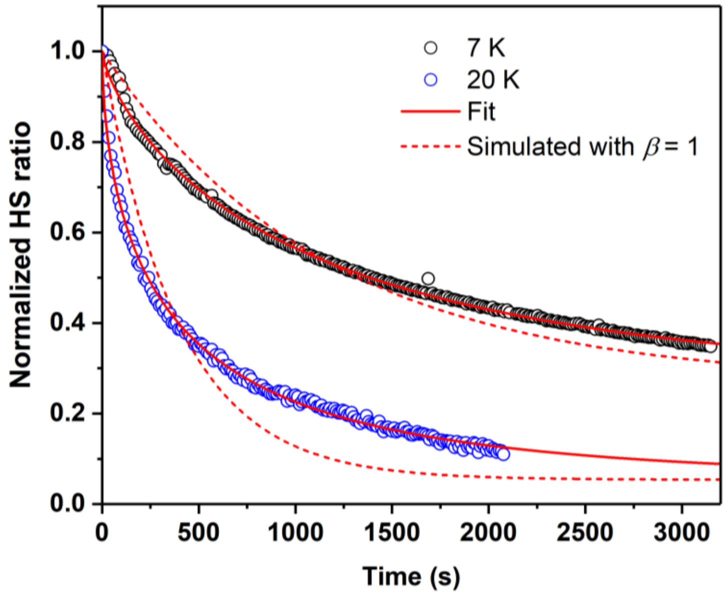

Supplementary Figure 19. Relaxation from the light excited state to the ground state at 7 and 20 K. The solid lines represent the fitting by stretched exponential law: $\gamma_{HS}(t) = (1-\gamma_0)\exp[-(t/\tau_0)^{\beta}] + \gamma_0$, where γ_0 is the ratio of remnant HS species, τ_0 is the characteristic relaxation time and β is the distribution parameter. The fitting relaxation times are 1158(29) s with a distribution parameter of 0.74(1) for 7 K and 392(8) s with a distribution parameter of 0.57(1) for 20 K. The dashed lines are simulated with the same parameters except that β is fixed by 1. It can be concluded that the short-time relaxation of this system is faster than that of the system with monodispersed relaxation time.

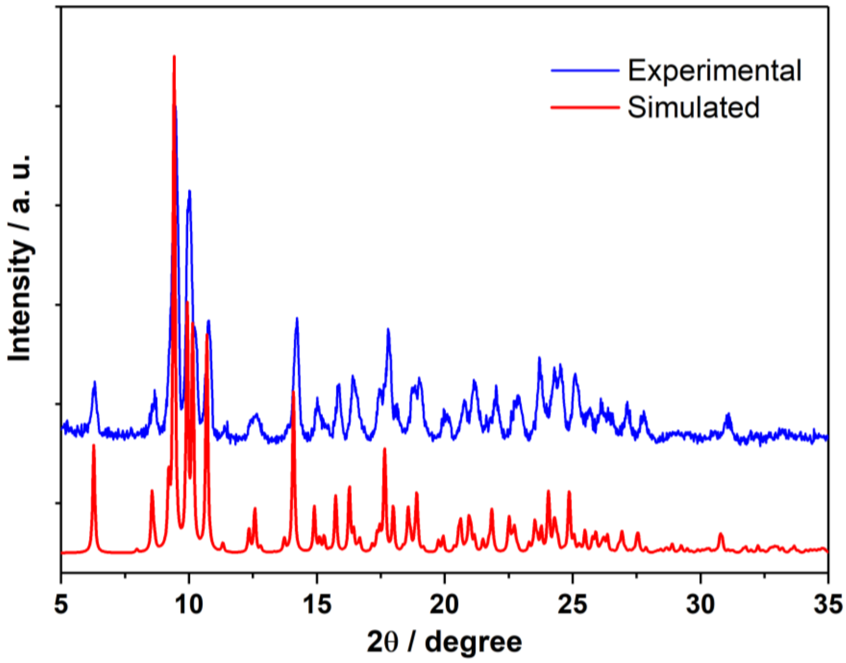

Supplementary Figure 20. Powder X-ray diffraction pattern of **1**·0.5EtOH at room temperature.

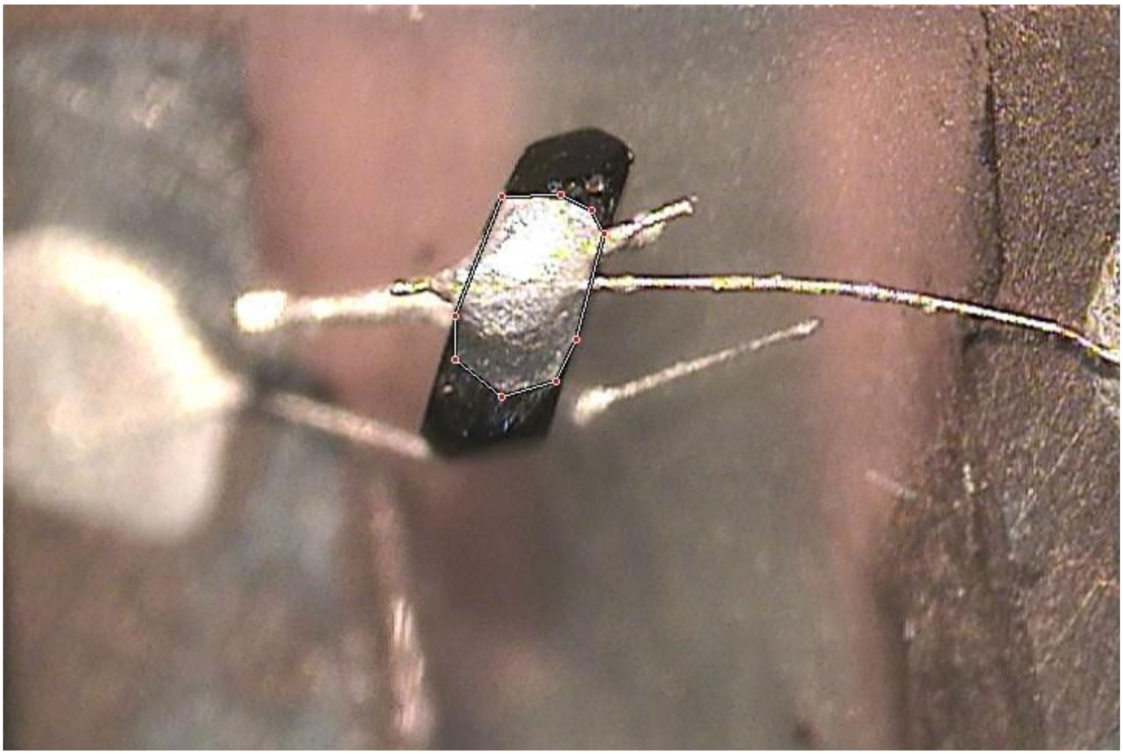

Supplementary Figure 21. The single crystal used in the pyroelectric measurements (the area covered with silver paste is about 0.22 mm^2).

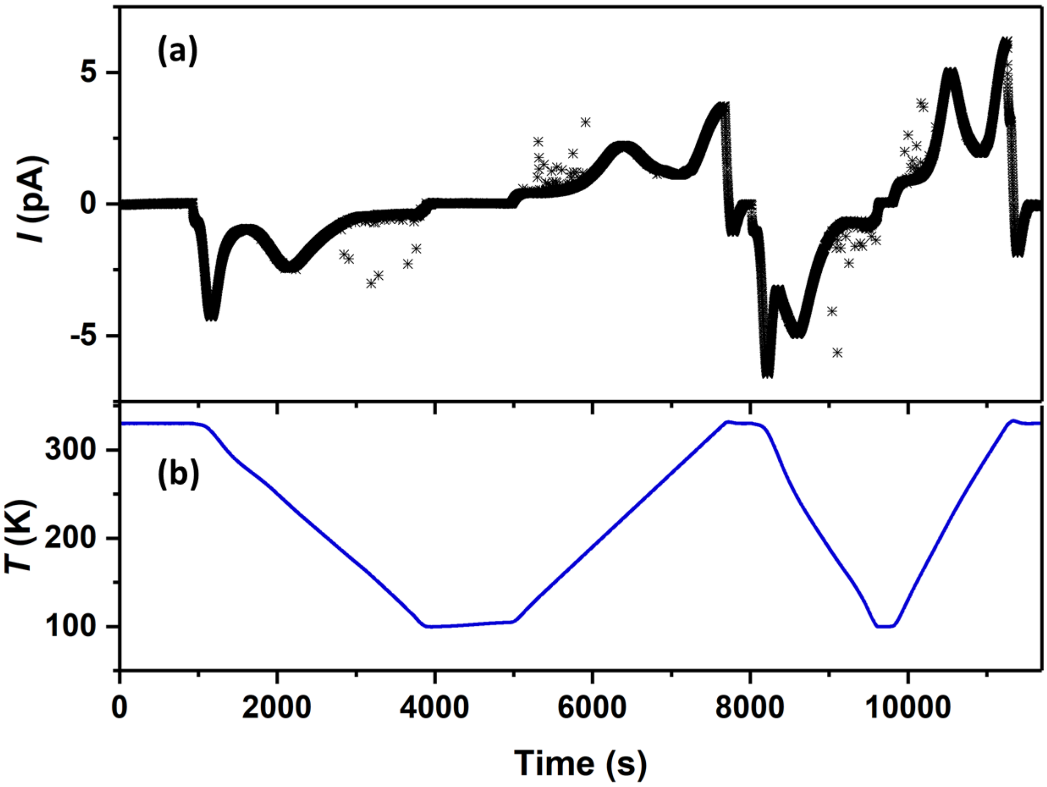

Supplementary Figure 22. Raw data of pyroelectric measurement in the MPMS-XL chamber ((a) and (b) panels show the pyroelectric current and temperature, respectively). In the experimental setup, the pyroelectric current of the single crystal was recorded at constant stress, and the corresponding pyroelectric coefficient (p^{σ}) can be further decomposed into two main terms, i.e., pyroelectric coefficient at constant strain (p^{ε}) and strain-induced pyroelectric coefficient, which are termed as the primary and secondary pyroelectric coefficients, respectively. The general relationship for p^{σ} is $p^{\sigma} = p^{\varepsilon} + dc\alpha$, where d, c, and α represent the piezoelectric, elastic stiffness and thermal expansion tensors, respectively.

Supplementary Table 5. Optimized coordinates for $[Co(SS\text{-cth})(phendiox)]^{+}$ in closed-shell singlet and quintet states.

Singlet	x	y	z	Quintet	x	y	z
Co	-1.171004	0.000076	0.000074	Co	-1.195657	0.000215	0.000039
O	0.171121	-1.043134	0.792331	O	0.415099	0.968994	-0.902541
O	0.171213	1.043069	-0.792319	O	0.415437	-0.968576	0.90288
N	-2.475551	1.362885	-0.800768	N	-2.554315	-1.48978	0.855193
N	-1.20765	-0.962721	-1.8004	N	-1.399507	0.948794	1.984929
N	-2.475715	-1.362429	0.80113	N	-2.555625	1.488369	-0.855313
N	-1.207091	0.962866	1.800516	N	-1.399085	-0.949173	-1.985013
C	1.38166	-0.543499	0.414148	C	1.554348	0.528115	-0.491125
C	1.381708	0.54325	-0.414244	C	1.554516	-0.527333	0.491434
C	2.601074	1.145137	-0.860392	C	2.806777	-1.060685	0.97787
C	2.585623	2.276854	-1.705065	C	2.796122	-2.10123	1.927882

C	3.764475	2.85627	-2.127889	C	3.978703	-2.631245	2.407775
C	4.993852	2.314909	-1.715574	C	5.198865	-2.125232	1.940473
C	5.025775	1.205918	-0.890959	C	5.224082	-1.101796	1.00459
C	3.84278	0.584112	-0.435348	C	4.039227	-0.540743	0.496311
C	3.842728	-0.584732	0.435045	C	4.039037	0.542186	-0.496216
C	5.025668	-1.206713	0.890562	C	5.223698	1.103531	-1.004621
C	4.993647	-2.315695	1.715184	C	5.198132	2.126948	-1.940517
C	3.764224	-2.856869	2.127606	C	3.977797	2.632649	-2.407709
C	2.585423	-2.277278	1.704877	C	2.795397	2.102341	-1.927694
C	2.600974	-1.145568	0.860196	C	2.806408	1.061813	-0.977664
C	-2.365141	1.147926	-2.270931	C	-2.432219	-1.259989	2.315153
C	-2.315495	-0.330303	-2.563216	C	-2.519205	0.229125	2.627594
C	-1.183018	-2.475421	-1.932278	C	-1.423481	2.449402	2.084119
C	0.209101	-2.98425	-1.537557	C	-0.002946	2.938865	1.76707
C	-1.43967	-2.8986	-3.394211	C	-1.796355	2.912554	3.505859
C	-2.290526	-3.067831	-1.046405	C	-2.450872	3.041926	1.092699
C	-2.209215	-2.811614	0.459192	C	-2.218711	2.883098	-0.420624
C	-3.176386	-3.750023	1.193412	C	-3.021039	3.945971	-1.182846
C	-2.365033	-1.147469	2.271274	C	-2.433604	1.258691	-2.315292
C	-2.314982	0.330756	2.563527	C	-2.519392	-0.230505	-2.627646
C	-1.181895	2.475565	1.932228	C	-1.421724	-2.449779	-2.084244
C	0.210224	2.983726	1.536653	C	-0.000798	-2.938044	-1.767186
C	-1.437631	2.899143	3.394212	C	-1.794197	-2.913287	-3.50599
C	-2.289557	3.068232	1.046738	C	-2.44856	-3.043211	-1.092814
C	-2.208702	2.812015	-0.458879	C	-2.216415	-2.884218	0.420481
C	-3.175883	3.750621	-1.192836	C	-3.017918	-3.947694	1.182744
H	-3.437922	1.161883	-0.522788	H	-3.522848	-1.316067	0.587111
H	-0.329046	-0.600864	-2.17552	H	-0.538572	0.629392	2.426707
H	-3.438087	-1.161223	0.5233	H	-3.523981	1.313927	-0.587067
H	-0.328515	0.600769	2.175473	H	-0.538396	-0.628952	-2.426677
H	1.62847	2.679203	-2.014335	H	1.838555	-2.473436	2.269876
H	3.744821	3.725482	-2.775531	H	3.964772	-3.431756	3.137977
H	5.922114	2.766968	-2.044761	H	6.132001	-2.534158	2.310041
H	5.989148	0.81402	-0.592006	H	6.185716	-0.739036	0.667732
H	5.989075	-0.81496	0.591528	H	6.185455	0.741016	-0.667852
H	5.921869	-2.767891	2.044298	H	6.13113	2.536102	-2.310184
H	3.744493	-3.726074	2.775255	H	3.963593	3.433145	-3.137922
H	1.628236	-2.679481	2.014231	H	1.837704	2.4743	-2.269606
H	-3.197185	1.603498	-2.811141	H	-3.199647	-1.792942	2.883688
H	-1.435678	1.623173	-2.584538	H	-1.454735	-1.643112	2.617294
H	-2.199967	-0.481724	-3.637763	H	-2.532301	0.363145	3.713554
H	-3.25305	-0.803941	-2.264204	H	-3.459899	0.633125	2.242278
H	0.465493	-2.729744	-0.515514	H	0.347744	2.573647	0.80368
H	0.245252	-4.068826	-1.65739	H	0.025801	4.030465	1.757355

H	0.97179	-2.553818	-2.192184	H	0.698093	2.598143	2.534727
H	-2.46032	-2.703283	-3.728594	H	-2.836799	2.693805	3.757458
H	-0.744956	-2.398526	-4.074672	H	-1.153262	2.433567	4.249618
H	-1.268964	-3.972565	-3.486992	H	-1.657894	3.991976	3.595688
H	-3.277941	-2.759186	-1.412961	H	-3.455387	2.670872	1.335101
H	-2.26074	-4.150316	-1.191626	H	-2.488735	4.113824	1.302135
H	-1.190386	-2.985604	0.801221	H	-1.158192	3.00363	-0.649673
H	-2.973246	-4.782445	0.903349	H	-2.733287	4.944326	-0.846814
H	-3.075004	-3.699036	2.277238	H	-2.845014	3.905419	-2.258456
H	-4.217566	-3.531765	0.930798	H	-4.095699	3.829783	-1.004581
H	-1.435637	-1.622934	2.584747	H	-1.4565	1.642649	-2.617618
H	-3.197104	-1.602832	2.811619	H	-3.201579	1.791015	-2.883674
H	-2.199247	0.482153	3.638052	H	-2.532505	-0.364592	-3.7136
H	-3.252465	0.804623	2.264652	H	-3.459729	-0.635212	-2.242193
H	0.973068	2.553211	2.191046	H	0.700035	-2.596484	-2.534658
H	0.466023	2.728768	0.514564	H	0.349414	-2.572803	-0.80364
H	0.246862	4.068328	1.656099	H	0.028913	-4.029621	-1.757775
H	-2.458193	2.704347	3.729163	H	-2.834818	-2.695427	-3.757624
H	-0.742782	2.398915	4.074423	H	-1.151486	-2.433785	-4.249749
H	-1.266413	3.973052	3.486688	H	-1.654834	-3.992597	-3.595786
H	-2.259477	4.150708	1.191958	H	-2.485507	-4.11514	-1.302276
H	-3.276929	2.759801	1.41359	H	-3.453407	-2.673004	-1.335149
H	-1.189927	2.985809	-0.801171	H	-1.155785	-3.003974	0.649438
H	-3.074794	3.699625	-2.276689	H	-2.841804	-3.907068	2.258338
H	-2.972467	4.783	-0.902816	H	-2.729513	-4.945835	0.846641
H	-4.217037	3.532562	-0.929953	H	-4.092682	-3.832253	1.004606

Supplementary Table 6. Coordinates of H-optimized models for *Δ*-[Co(phendiox)(*SS*-cth)](ClO_4) and *Λ*-[Co(phendiox)(*RR*-cth)](ClO_4) at 123 K.

Δ-Co	x	y	z	*Λ*-Co	x	y	z
Co	0.327411	0.051125	0.031197	Co	-0.302717	0.053917	-0.011889
O	-0.992567	-0.985525	-0.835907	O	1.036025	-0.936789	-0.916638
O	-1.056022	1.131766	0.729887	O	1.070679	1.131662	0.717216
N	1.621956	-1.302284	-0.674432	N	-0.207947	-0.94264	1.73361
H	2.557853	-1.123239	-0.299195	H	0.679623	-0.5733	2.075048
N	1.598322	1.363717	0.861095	N	-1.595297	1.302731	0.874509
H	2.564737	1.166094	0.579145	H	-2.557055	1.014879	0.669773
N	0.280435	-0.875726	1.819051	N	-1.594728	-1.273491	-0.754685
H	-0.585557	-0.470556	2.176232	H	-2.547303	-1.0977	-0.42213
N	0.521534	0.99185	-1.756179	C	3.426669	-2.305926	-1.671206
H	-0.326989	0.633599	-2.195599	H	2.464075	-2.674322	-2.003842
C	-4.70014	0.561783	0.31129	N	-0.4973	1.060718	-1.753988
C	1.670099	-1.11041	-2.143926	H	0.368579	0.750302	-2.196483
H	0.778028	-1.578279	-2.562513	C	3.451713	-1.152928	-0.859196

H	2.563658	-1.569104	-2.564808	C	4.697222	-0.619845	-0.425928
C	-2.201346	-0.493785	-0.480636	C	2.240142	-0.489713	-0.48411
C	-5.905544	1.15347	0.749845	C	5.868786	-1.305173	-0.812879
H	-6.849319	0.723635	0.438532	H	6.833075	-0.942642	-0.480518
C	1.352018	2.814295	0.555215	C	-1.407666	1.081894	2.338721
H	0.31603	2.995663	0.838652	H	-2.251435	1.478164	2.9035
C	1.327611	-2.752594	-0.369765	H	-0.495531	1.604011	2.62958
H	0.341465	-2.936986	-0.794861	C	3.494616	2.318142	1.61196
C	1.409737	-0.313704	2.607964	H	2.545587	2.710783	1.95355
H	2.331971	-0.805094	2.304254	C	4.588952	-2.933581	-2.053203
H	1.2624	-0.472556	3.676331	H	4.553691	-3.814657	-2.683857
C	-3.486117	1.165413	0.746926	C	3.489455	1.168592	0.779996
C	-4.656394	-0.60933	-0.540116	C	1.207476	-2.937712	1.332971
C	-3.34453	-2.310577	-1.716831	H	1.37702	-2.705701	0.288797
H	-2.369612	-2.696377	-1.986803	H	1.279089	-4.018595	1.471182
C	1.551563	3.085211	-0.917421	H	2.011434	-2.476393	1.912696
H	2.561873	2.7692	-1.198104	C	5.89214	2.425485	1.454012
H	1.53264	4.16841	-1.062247	H	6.822074	2.929863	1.690494
C	1.503362	1.152655	2.326177	C	2.257021	0.599016	0.344875
H	0.604347	1.668278	2.668166	C	4.718264	0.600489	0.353046
H	2.370084	1.570433	2.840129	C	5.824268	-2.434772	-1.606108
C	0.199258	-2.376729	1.959849	H	6.745185	-2.933251	-1.885537
C	-2.233815	0.598772	0.325784	C	-2.397161	-3.619784	-1.13882
C	-0.885434	3.007338	-1.639691	H	-2.23329	-4.658873	-0.846279
H	-1.579828	2.580931	-2.36972	H	-2.360134	-3.584124	-2.227329
H	-0.911212	4.093263	-1.756097	H	-3.398086	-3.319053	-0.821018
H	-1.240195	2.746316	-0.647993	C	-1.477178	2.770118	0.578679
C	2.365396	-3.656545	-1.028332	H	-0.469727	3.044577	0.89092
H	2.30898	-3.637938	-2.115998	C	-0.150467	-2.458732	1.816597
H	2.199422	-4.689701	-0.715456	C	-1.291597	-0.38802	2.587182
H	3.377767	-3.368383	-0.732316	H	-1.090583	-0.567336	3.643778
C	0.949612	2.920666	-3.304112	H	-2.228645	-0.875883	2.325331
H	1.99797	2.710085	-3.517532	C	-1.300905	-3.042936	0.997967
H	0.805589	3.998124	-3.404241	H	-1.242024	-4.1288	1.105623
H	0.331515	2.437519	-4.066553	H	-2.262684	-2.758622	1.438272
C	2.284527	3.724997	1.367404	C	5.908668	1.287084	0.689902
H	2.086583	3.684064	2.437999	H	6.858539	0.910718	0.332366
H	2.136694	4.759868	1.052056	C	-1.61012	-1.048148	-2.214385
H	3.33203	3.463347	1.192726	H	-2.485203	-1.515635	-2.666533
C	1.307813	-3.012639	1.116701	H	-0.704667	-1.499507	-2.622362
H	2.287109	-2.748015	1.529529	C	4.681994	2.926237	1.946981
H	1.219045	-4.093699	1.255567	H	4.680068	3.810454	2.575138
C	-3.40423	-1.14424	-0.928847	C	-0.928729	3.021681	-3.281612
C	-5.920792	2.251882	1.549471	H	-0.816808	4.105505	-3.348034

H	-6.867374	2.679119	1.861468	H	-1.954941	2.780059	-3.559934
C	0.420812	-2.789601	3.426875	H	-0.248251	2.575577	-4.012278
H	1.443487	-2.625807	3.766052	C	-1.638753	0.418985	-2.48059
H	0.21091	-3.856366	3.525635	H	-2.572145	0.845535	-2.110611
H	-0.263788	-2.259063	4.095089	H	-1.577991	0.607166	-3.552661
C	1.686369	0.361006	-2.431269	C	0.790654	3.157865	-1.503262
H	2.603538	0.80324	-2.041787	H	0.747687	4.24825	-1.554726
H	1.658315	0.529174	-3.508447	H	1.542715	2.824619	-2.225042
C	0.528358	2.49873	-1.891503	H	1.125741	2.856361	-0.515311
C	-1.191344	-2.840876	1.538067	C	-0.569804	2.578818	-1.847356
H	-1.962396	-2.337289	2.127819	C	-1.659887	3.069252	-0.895641
H	-1.284914	-3.913889	1.717734	H	-1.705423	4.156012	-1.004443
H	-1.384337	-2.639539	0.491072	H	-2.642381	2.703974	-1.212201
C	-4.492664	-2.925965	-2.142562	C	-1.326696	-2.7362	-0.481487
H	-4.435118	-3.81823	-2.756462	H	-0.34993	-2.932003	-0.921542
C	-5.817614	-1.290587	-0.997636	C	-0.305245	-2.91333	3.274632
H	-6.79724	-0.918985	-0.726511	H	-0.118476	-3.987425	3.330203
C	-4.716269	2.84622	1.984393	H	-1.304078	-2.736644	3.674263
H	-4.740342	3.719801	2.625456	H	0.425539	-2.420818	3.922545
C	-3.512441	2.297489	1.588617	C	-2.512859	3.566999	1.388394
H	-2.56955	2.725012	1.905271	H	-3.522758	3.192806	1.196663
C	-5.726466	-2.403234	-1.791224	H	-2.479987	4.615749	1.086552
H	-6.634642	-2.890072	-2.129864	H	-2.324383	3.535203	2.460761
Cl	4.998404	-0.147695	-0.056855	Cl	-5.071605	-0.173229	0.056808
O	6.374676	-0.211636	0.304452	O	-6.393223	-0.366158	0.515364
O	4.206965	-0.829695	0.936626	O	-4.943534	1.041701	-0.665461
O	4.772815	-0.790574	-1.316287	O	-4.74557	-1.24126	-0.829867
O	4.554297	1.217773	-0.138594	O	-4.17739	-0.159721	1.118633
